# Flavonoids and Their Role in Preventing the Development and Progression of MAFLD by Modifying the Microbiota

**DOI:** 10.3390/ijms252011187

**Published:** 2024-10-17

**Authors:** Aneta Sokal-Dembowska, Sara Jarmakiewicz-Czaja, Rafał Filip

**Affiliations:** 1 Institute of Health Sciences, Medical College of Rzeszow University, 35-959 Rzeszow, Poland.; sjczaja@ur.edu.pl (S.J.-C.); 2 Institute of Medicine, Medical College of Rzeszow University, 35-959 Rzeszow, Poland; 3 Department of Gastroenterology with IBD Unit, Clinical Hospital No. 2, 35-301 Rzeszow, Poland

**Keywords:** antioxidants, flavonoids, inflammation, liver disease, microbiota

## Abstract

With the increasing prevalence and serious health consequences of metabolic-associated fatty liver disease (MAFLD), early diagnosis and intervention are key to effective treatment. Recent studies highlight the important role of dietary factors, including the use of flavonoids, in improving liver health. These compounds possess anti-inflammatory, antioxidant, and liver-protective properties. Flavonoids have been shown to affect the gut microbiota, which plays a key role in liver function and disease progression. Therefore, their role in preventing the development and progression of MAFLD through modulation of the microbiome seems to be of interest. This narrative review aims to consolidate the current evidence on the effects of selected flavonoids on MAFLD progression, their potential mechanisms of action, and the implications for the development of personalized dietary interventions for the management of liver disease.

## 1. Introduction

Liver disease is responsible for approximately 2 million deaths annually and approximately two thirds of all liver-related deaths occur in men [1]. The most common causes of chronic liver disease are hepatitis B and C virus infection, autoimmune diseases, alcohol consumption, steatosis associated with metabolic dysfunction (MAFLD), and cirrhosis, which is the final stage of various dysfunctions of this organ [2]. In addition, polymorphisms of certain genes can affect the occurrence of liver fibrosis, e.g., PNPLA-3 and MBOAT7 [3]. MAFLD accounts for 50% of cases of chronic liver disease [4] and cirrhosis is the 11th leading cause of death worldwide [5]. Chronic liver disease is characterized by progressive deterioration of liver function [6]. Therefore, the therapeutic approach is largely dependent on the degree of organ function and not only the type of disease itself [5]. Given the magnitude of the problem and the prognosis associated with chronic advanced liver disease and hepatocellular carcinoma, early diagnosis appears to be very important for the rapid implementation of appropriate treatment [7]. As MAFLD may become one of the most important chronic liver diseases, it is important to understand the phenotypes of the disease in order to be able to implement effective, individualized therapeutic therapies in individual patients [8].

Modern therapeutic approaches, depending on the cause of the disease, include drug therapies, including antivirals and immunosuppressants, and surgical treatment if liver transplantation is required. However, the critical importance of lifestyle changes in the prevention and treatment of liver disease cannot be overlooked [5]. Current nutritional recommendations focus primarily on preventing malnutrition and sarcopenia. Therefore, ensuring an adequate supply of protein and energy in patients’ diets is considered a cornerstone. Another important element is an adequate intake of fruits and vegetables in the diet [9], which are a source of valuable compounds, such as phytonutrients, that have protective, antioxidant, and anti-inflammatory effects [10,11]. Consumption of vegetables in general has been shown to be associated with a lower risk of liver cancer. In turn, increased consumption of cruciferous vegetables and legumes, among others, has been associated with a lower risk of mortality from chronic liver disease [12].

Widespread in nature, flavonoids are known for their many valuable biological properties [13] and their role in the treatment of MAFLD has been increasingly discussed by researchers in recent years. Flavonoids with potential beneficial effects on liver health include quercetin, rutin, luteolin, epigallocatechin-3, silibinin, puerarin naringenin, apigenin, genistein, and resveratrol, among others [14].

Previous data from the National Health and Nutrition Examination Survey (NHANES) and the Food and Nutrient Database for Dietary Studies have already suggested that using flavonoids offers a potential opportunity to prevent MAFLD [15]. Flavonoids may be a promising therapeutic component in the treatment of MAFLD and its complications, as a recent meta-analysis of randomized controlled trial data showed that flavonoids improve MAFLD through beneficial effects on liver function, lipid profile, and inflammation [16]. A flavonoid-rich diet was associated with a lower risk of MAFLD and lower imaging biomarkers of MAFLD in a prospective cohort study by Bell et al. [17].

In addition to anti-inflammatory or antioxidant effects, they can exert beneficial effects on the intestinal microbiota, modulating it by promoting or inhibiting specific microbial species in the intestinal tract, and modifying their metabolites [13]. Flavonoids may contribute to an increase in the relative abundance of intestinal probiotics such as *Bifidobacterium* and *Lactobacillus* while decreasing the relative abundance of *Lachnoclostridium* or *Bilophila* [18,19] In addition, they exert a protective effect on the intestinal epithelial barrier through the production of short-chain fatty acids (SCFAs) or lithocholic acid [20]. According to current evidence, flavonoids are characterized by their ability to inhibit lipid metabolism disorders and therefore can have an effect on reducing oxidative stress, inflammation, and disrupting the intestinal microbiota [13]. Flavonoids possess potent pharmacological activity, mitigating the effects of MAFLD and metabolic dysfunction-associated steatohepatitis (MASH). This is likely related to the effects of flavonoids on the gut microbiota, regulation of lipid metabolism, autophagy, oxidative stress, and inflammation in the body [21].

On this basis, this narrative review aims to update reports on the effects of selected flavonoids with potential applications in the treatment of chronic liver disease. The tables include detailed findings from studies over the past 5 years that analyzed the simultaneous effects of individual flavonoids on liver health, metabolic status, inflammation, and gut microbiota.

### 1.1. Chronic Liver Disease Pathogenesis

Hepatitis, steatosis, or cholestatic liver diseases cause continuous damage to liver cells, leading to organ fibrosis, its failure, and cirrhosis [22,23]. It has been shown that the onset and regression of fibrosis through various pathways is multidirectional and nonunilateral, for example, changing the MMP/TIMP-1 ratio and accelerating ECM degradation, inhibiting the TGF-β1 signaling pathway, reducing IL-6 and types I and III collagen expression [23]. The result of cellular injury is a naturally occurring process of the healing of damaged tissue, during which fibrogenic pathways are activated. The production of myofibroblasts (MFBs) that promote scar formation by activated hepatic stellate cells (HSCs) occurs [24]. Various cells, including Kupffer cells (KCs), hepatocytes, and numerous signaling pathways, can regulate HSC activation and are potential therapeutic targets that can influence the reversal of fibrosis [25]. For repair, fibrogenic components of the extracellular matrix (ECM) are secreted into the Disse space. During organ damage, the composition and density of the ECM are altered [26].

### 1.2. The Role of Inflammation and Oxidative Stress in Disease Progression

Damage to hepatocytes results in the release of threat-associated molecular patterns (DAMPs) or, in the case of infection, pathogen-associated molecular patterns (PAMPs). Both DAMPs and PAMPs signal to pattern recognition receptors (PRRs) located on the surface of non-parenchymal cells and resident resistance cells. It is the activation of these cells through the interaction of PRRs and the aforementioned patterns that is responsible for the production of cytokines and chemokines that promote inflammation [24]. In addition, the inflammatory response can be sustained by the excessive production of reactive oxygen species (ROS). ROS can affect the activation and proliferation of HSCs, thus initiating and exacerbating fibrosis [25]. They are also responsible for the apoptosis and necrosis of hepatocytes, leading to the release of inflammatory mediators including transforming growth factor (TGF)-β and tumor necrosis factor (TNF)-α. Furthermore, they stimulate KCs to produce profibrogenic mediators and accelerate the recruitment of circulating inflammatory cells to the liver [26].

### 1.3. Intestinal Microbiota Disorders in the Development of Liver Diseases: Gut–Liver Axis

It has been shown that the gut–liver axis is essential for maintaining the normal physiological state of the body and plays an important role in the prognosis of many diseases, including in patients with MAFLD [27]. Liver diseases, including MAFLD and MASH, have been associated with intestinal dysbiosis and small intestinal bacterial overgrowth (SIBO) [28]. Yang et al. identified the characteristic microbial taxa of patients with MAFLD. They observed that a decrease in *Alistipes* is characteristic of this group of patients and that this decrease is negatively associated with glucose, gamma-glutamyltransferase (GGT), and alanine aminotransferase (ALT) levels. On the contrary, *Dorea*, *Lactobacillus*, and *Megasphaera* were enriched in the MAFLD group [29]. An analysis by Qi et al. showed a decrease in *Bacteroidetes* in patients with liver disease, including MAFLD and cirrhosis [30]. According to Boicean et al., fecal microbiota transplantation may become one of the treatments for cirrhosis [31]. Promising results in the treatment of MAFLD have also been associated with the use of rifaximin, prebiotics, probiotics, and glucagon-like peptide 1 (GLP-1) agonists [32]. However, well-designed clinical trials in this area are still lacking.

Changes in the microbiota and thus a decrease in intestinal mucosal epithelial barrier permeability and changes in metabolite release can promote liver regeneration. The gut microbiota can modulate the release of inflammatory factors including interleukin (IL)-6, interferon (IFN)-γ, TGF-β, TNF-α, and hepatocyte growth factor (HGF), thus regulating the immune microenvironment of the liver [33]. The gut microbiome can control hepatocytes via Gram-negative bacteria/lipopolysaccharide (LPS), which interacts with toll-like receptors (TLRs), especially TLR4, in KCs and HSCs [34].

Intestinal dysbiosis can lead to impaired gut–liver circulation, the action of farnesoid X receptor (FXR) agonists, and the production of BA itself [35]. Increased levels of total fecal BA, including cholic acid and chenodeoxycholic acid, are observed in patients with MAFLD [32]. In addition, similar changes are observed in patients with cirrhosis. Cholic acid and chenodeoxycholic acid are 7α-dehydroxylated exclusively by *Clostridium* bacteria, the amount of which is reduced due to reduced substrate levels. This leads to reduced conversion of primary BA to secondary BA in more pathogenic families such as *Enterobacteriaceae*, causing dysbiosis [36].

Recent evidence suggests that targeting the gut microbiota may help treat and mitigate the progression of liver disease. Thus, the microbiota taxa may be important therapeutic targets in this patient population.

## 2. The Effect of Flavonoids on Chronic Liver Diseases through the Modulation of Gut Microbiota

The available scientific data suggest that flavonoids may improve liver health by reversing the adverse effects associated with dysregulation of the gut microbiota. Flavonoid supplementation has been shown to increase SCFA production, reduce the *Firmicutes*/*Bacteroidetes* ratio, improve the intestinal barrier, and inhibit the growth of harmful bacteria [21]. Through interactions with the intestinal microbiota, flavonoids may influence lipid metabolism by modulating BA excretion and promoting the growth of microorganisms that play a positive role in lipid metabolism, such as *Akkermansia muciniphila*, *Bifidobacterium*, and *Lactobacillus*. Reducing lipogenesis and genes related to inflammation and increasing the expression of genes related to fatty acid oxidation and fatty acid oxidase activity may lead to a reduction in fat accumulation and weight gain [13].

### 2.1. Silymarin

Silymarin is a mixture of polyphenolic molecules, more precisely flavonolignans, among which we can distinguish silibinin A, silibinin B, isosilibin A, isosilibin B, silicristin, isosilicristinm, the flavonoid tacphylline, and silidanin [37], an extract of spotted thistle that is considered a hepatoprotective compound [38]. Existing data highlight the role of the gut microbiota in the hepatoprotective effects of silymarin on the liver. However, it is still the case that most of the scientific evidence comes from animal model studies (Table 1) [32,33,34,35].

#### 2.1.1. Effect of Silymarin on Liver Metabolic Status, Inflammation, and Gut Microbiota

Silymarin, due to its antioxidant properties, has been demonstrated to attenuate lipid peroxidation and free radical production. [38]. Moreover, it has been shown to have anti-inflammatory and anti-fibrotic effects [39]. Silybin has also shown promise in lowering glycemia and improving insulin sensitivity through peroxisome proliferator-activated receptor (PPAR)-γ activation [40,41]. In addition, silymarin may stimulate the endogenous production of antioxidants such as glutathione (GSH), catalase (CAT), glutathione peroxidase (GPX), and superoxide dismutase (SOD), and reduce inflammation by inhibiting the nuclear factor (NF)-κB and lowering TNF-α levels [40].

In an animal model study, Wang et al. confirmed that silymarin or silymarin with the salvianolic acid B and puerarin formula improved liver steatosis induced by a high-fat diet (HFD). Supplementation with these compounds improved liver function, including lowering pro-inflammatory cytokines and enabling more cholesterol to be metabolized to bile acids in the liver. Additionally, improvements in changes in the intestinal ecosystem contributed to increased SCFA production, which relieved MAFLD [42]. Wen-Long et al. also showed that silymarin supplementation contributed to microbiological changes. In addition, the study authors observed that these changes were associated with increased vitamin B12 production, which was associated with improved lipid metabolism and decreased pro-inflammatory cytokines [43]. Improvements in liver function were also observed by Guo et al. in a study conducted in laying hens. Supplementation of the diet with silymarin at a dose of 500 mg/kg resulted in a significant reduction in alanine aminotransferase (ALT) and aspartate aminotransferase (AST) levels and improved the lipid metabolism of the cecal microbiota function. Therefore, it is likely that silymarin can alleviate bile acid stasis and increase the ability of metabolic microbes to metabolize bile acids [44]. Ralli et al. showed that silymarin can also potentiate the effects of probiotics alone in the treatment of MAFLD. In combination with piperine, fulvic acid, and probiotics, silymarin can significantly reduce liver steatosis by modulating the microbiota [45].

The first population-based study by Jin et al. showed that silymarin supplementation at a dose of 103.2 mg silibin per day for 24 weeks could have a significant protective effect against liver stiffness in patients with MAFLD by modulating the microbiota. Analysis of the gut microbiome showed that silymarin supplementation effectively modulated the composition and abundance of microbial populations [46].

Furthermore, results from a randomized placebo-controlled trial by Anushiravani et al. showed that silymarin supplementation at a dose of 140 mg/d for 3 months significantly reduced BMI and waist circumference in patients with MAFLD. Improvements in TGs, HDL, LDL levels, and the liver enzymes ALT and AST were also observed [47].

##### Mediterranean Diet Containing Flavonolignans in the Treatment of MAFLD

There has been evidence of a negative association between adherence to the Mediterranean diet and the degree of liver damage [48]. This dietary model is characterized by the consumption of foods rich in polyphenolic compounds, such as vegetables, fruit, wholegrain cereals, olive oil, nuts, and red wine [49]. The use of a hypocaloric Mediterranean diet (MED) combined with supplementation with hepatoactive compounds, including silymarin, in overweight/obese patients for 3 months resulted in significant improvements in nutritional status, body weight composition, biochemical parameters, and degree of liver steatosis (LS) compared to the baseline. The supplementation group showed a significantly greater improvement in liver steatosis [50]. The results of a randomized controlled trial by Abenavoli et al. showed that an MED with an antioxidant complex containing milk thistle extracts can improve anthropometric parameters but can also reduce insulin resistance and fat accumulation in the liver [51].

The use of the MED is known to have a beneficial effect on the gut microbiota, as it contains many substrates necessary for the conversion of bioactive compounds. In addition, the MED diet can be tailored and personalized based on the individual’s microbiome profile for optimal results [52].

This dietary model has applications in the treatment of chronic liver disease. A cross-sectional analysis of the RaNCD cohort showed that adherence to a MED diet, characterized by a high intake of fruits, vegetables, whole grains, legumes, nuts, and also fish, was associated with a lower risk of liver fibrosis in patients with MAFLD [53].

**Table 1:** Simultaneous effects of silymarin on the liver, metabolic state, inflammation, and gut microbiota.

Name of the Active Compound	Results of the Study	Reference
Animal studies		
Silymarin	reduced accumulation of lipid droplets in the liver, reduced levels of liver TGs and serum TC; improved glucose tolerance; improvement in infulino resistance; decreased levels of the liver inflammatory cytokines TNF-α and IL-6; increase in *Akkermansia* and *Blautia*; inhibition of proliferation of *Lactobacillus*, *Bacteroides*, *Clostridium*, and *Ileibacterium*.	Wang et al. (2024) [42]
	improving liver function, lowering ALT and AST levels; lowering levels of the liver inflammatory cytokines TNF-α and IL-6; improving lipid metabolism, preventing accumulation of TGs and TC accumulation; increase bacterial richness, increase ASV5 (*Akkermansia muciniphila*).	Sun et al. (2023) [43]
	improving liver function, lowering ALT and AST levels; decreased expression of genes related to lipid metabolism FXR, CYP7A1, BSEP, and MRP2; significant reduction in TG and TC levels and lower serum LDL-C and HDL-C; decreased endogenous bile acid synthesis and accelerated enterohepatic circulation; increased expression of the bile acid receptor FXR and decreased expression of CYP7A1; increasing the abundance of *Flavonifractor*; improving the relative abundance of the genus *Phocaeicola*.	Guo et al. (2024) [44]
Studies involving humans		
	a significant decrease in liver stiffness measurement from baseline; a significant difference in the change in serum GGT levels was found between the two groups (silymarin group and placebo) at 24 weeks; no significant changes in mean CAP, serum ALT and AST levels, AST/ALT ratio, total bilirubin concentrations, or APRI or FIB-4; AST/ALT, ApoA1, and SOD concentrations, ApoB levels significantly improved; no statistically significant differences in the concentrations of TC, TGs, HDL-C, LDL-C, and ApoA1 and the ratio of ApoA1/ApoB between the groups; no statistically significant differences in fasting blood glucose and insulin concentrations, HOMA-IR, and UA, SOD, and hsCRP concentrations before and after intervention between the two groups; no statistical differences in physical parameters between the groups (DBP, SBP, BMI, WHR, BF% and BMR); the abundance of *Selenomonadaceae* in the silymarin group decreased and *Oscillospiraceae* were significantly enriched.	Jin et al. (2024) [46]

Abbreviations: AST—aspartate aminotransferase, ALT—alanine aminotransferase, hsCRP—high-sensitivity C-reactive protein; LPS—lipopolysaccharide; MDA—malondialdehyde; GSH—glutathione; TC—total cholesterol; TG—triglycerides; HDL-C—high-density lipoprotein; LDL-C—low-density lipoprotein cholesterol; TNF-α—tumor necrosis factor-α, IL-6—interkulin-6; MPO—myeloperoxidase; HFD—high-fat diet.

### 2.2. Genistein

Genistein is an isoflavone widely distributed in legumes, especially in soybeans, but also in broccoli, cauliflower, and sunflower [54]. It is known for its antioxidant, anticancer, and cardioprotective properties. Genistein is used in the treatment of menopause due to its estrogenic effects. It is also considered an important modulator of various types of signaling pathways at the translational and transcriptional levels [55]. There are also data suggesting that genistein may reduce lipid accumulation, inflammation, insulin resistance, liver steatosis, and even liver fibrosis [56]. Furthermore, genistein supplementation has been associated with changes in the gut microbial community [57].

Obesity and visceral fat accumulation promote the development of both type II diabetes and MAFLD [58]. As current data indicate, the common characteristics of the aforementioned metabolic disorders are intestinal dysbiosis and chronic inflammation [59,60,61].

#### Effect of Genistein on Liver, Metabolic Status, Inflammation, and Gut Microbiota

Hou et al. showed that dietary genistein modulates homeostasis in the ageing gut and extends the health and life span of ageing mammals [62]. There are also data supporting the anti-inflammatory properties of genistein, although mostly from in vivo and in vitro studies. Genistein can exert such effects on the body, among other things, by inhibiting prostaglandins, nitric oxide synthase (iNOS), NF-κB or ROS, and scavenging free radicals [63]. Ortega-Santos et al. reached an interesting conclusion, investigating how genistein supplementation combined with a high-fat diet and exercise can affect inflammation and the gut microbiota. The intervention had a significant effect on increasing the abundance of *Ruminococcus*. Total levels of fecal bile acid and secondary fecal bile acid were significantly higher during exercise and as a result of the combination of exercise and supplementation with genistein. In addition, combining supplementation and exercise helped prevent the negative effects of HFD and combined treatment resulted in lower serum IL-6 concentrations [57].

A high-fat diet not only causes obesity. It can also increase intrahepatic lipid accumulation and disrupt the integrity of the intestinal barrier [64]. It is likely that genistein has protective effects against high-fat diet-induced insulin resistance and liver steatosis also by maintaining glucose homeostasis through modulation of the insulin signaling pathway and liver energy status [65]. In a study by Guevara-Cruz et al., two months of genistein supplementation at a dose of 50 mg/day reduced insulin resistance in obese subjects, accompanied by a reduction in gut microbiome dysbiosis and metabolic endotoxemia. In particular, an increase in the type of *Verrucomicrobia* and in particular the abundance of *Akkermansia muciniphila* were observed [66]. Supplementation of mice with ulcerative colitis (UC) with genistein accelerated SCFA production (at doses of 20 mg/kg and 40 mg/kg for 10 days). Inhibition of weight loss, increased colon length, increased expression of mucus components, and decreased disease activity index values were also observed [67].

SCFAs are known for their ability to modulate inflammation and immune responses [68]. Furthermore, they play an important role in protecting the intestinal barrier by inhibiting IL17 production and promoting IL-10 production [68,69]. The supply of genistein to mice consuming a normal diet resulted in better glucose tolerance and greater expression of *UCP1* and *PGC1α* in white adipose tissue compared to mice that received no supplementation. An enrichment of the microbiota with bacteria of the genus *Blautia*, *Ruminiclostridium*_*5*, and *Ruminiclostridium*_*9* was also observed. Interestingly, changes in the microbiota were correlated with markers of browning of adipose tissue and glucose tolerance. On the contrary, in obese mules, genistein supplementation alleviated the effects of HFD and increased the expression of *UCP1* and *PGC1α* expression in brown adipose tissue [70]. A study by Ahmed et al. showed that low BAT activity was associated with greater visceral fat accumulation, but did not observe that this process was mediated by the gut microbiota [71].

A meta-analysis of 12 randomized clinical trials confirmed that genistein supplementation can significantly increase high-density lipoprotein (HDL-C) and lower serum levels of fasting blood glucose, insulin, triglycerides (TGs), and homocysteine [72]. In a randomized clinical trial by Neshatbini Tehran et al., the administration of genistein in combination with other isoflavones (genistin, daidzin, daidzein, glycitin, glyciteini) for 12 weeks at a dose of 100 mg/d significantly reduced TGs, LDL, TC, and waist and hip circumference in patients with MAFLD [73]. Possibly, genistein can improve liver lipid metabolism by activating the estrogen receptor β and further modulating Akt/mTOR signaling. Therefore, it may have applications in the prevention and treatment of hepatic steatosis in postmenopausal women [74]. Pummoung et al., in an animal model study, showed that MASH can be worsened by estrogen deficiency and genistein supply can alleviate liver steatosis and apoptosis by decreasing PPARγ expression and increasing adiponectin expression [75].

**Figure 1:**
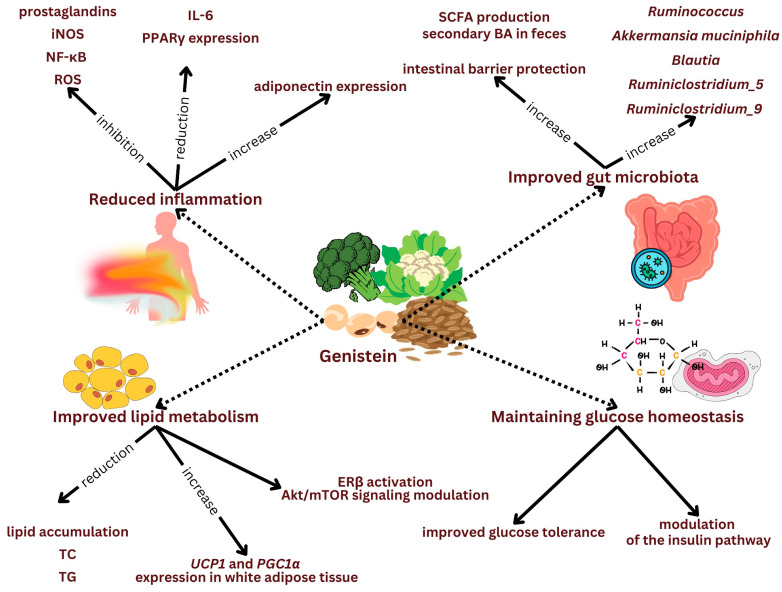
The potential effect of genistein in mitigating MAFDL and preventing its further development. Genistein exerts its effect through several mechanisms. It may influence the improvement of the intestinal microbiota, helps maintain proper glucose homeostasis, improves lipid metabolism, and contributes to reducing inflammation in the body.

The results of available research on genistein supply for the prevention and treatment of chronic liver disease are promising. However, there is still a lack of data that examine the effects of its supply simultaneously on liver health, inflammation, metabolic status, and microbiota composition. This potential impact is shown in Figure 1.

### 2.3. Narginin and Naringenin

Naringin (NAR) and its aglycone form naringenin belong to the flavones from the flavonoids group and are mainly found in citrus fruits, including lemon, tangerine, grapefruit, or orange, as well as bergamot and tomatoes. Numerous articles point to their potential anticancer and neurodegenerative disease risk reduction effects [76,77]. The results of an exploratory randomized clinical trial conducted by Notarnicola et al. showed that orange consumption may be helpful in the adjunctive treatment of MAFLD. Consumption of oranges at 400 g per day for 4 weeks led to a reduction in hepatic steatosis and a decrease in plasma gamma glutaryl transferase [78].

Recently, the use of these substances in alleviating the symptoms of COVID-19 has also been studied due to their anti-inflammatory and antiviral properties [79].

#### Effect of Narginin and Naringenin on Liver, Metabolic Status, Inflammation, and Gut Microbiota

Currently available data suggest that NAR can attenuate the activity of ulcerative colitis, among other things, by reducing the secretion of pro-inflammatory cytokines (TNF-α, IL-6 and IL-1β), activating PPARγ, and inhibiting NF-κB. Similar effects have been shown to improve MAFLD. NAR supply can result in improved lipid profile parameters and reduced liver inflammation (inhibition of IL-1β, IL-6, IL-12, TNF-α, and IFN-γ). NAR has also been shown to inhibit NF-κB and MAPK signaling pathways in both UC and MAFLD [80].

The supply of NAR in mice fed HFD diets in a study by Mu et al. attenuated HFD-induced hepatic lipid accumulation and weight gain and affected the structure of the gut bacterial community [81]. Similar results were obtained by Yu et al. with the use of naringenin. The authors of the study concluded that it could effectively alleviate liver and intestinal inflammation in the course of MAFLD. The results of the study showed that naringenin can effectively reduce MASH by improving steatosis, inflammation, and swelling of liver cells. In addition, it can protect the intestinal barrier by regulating tight junction proteins [82]. According to Cao et al., the supply of naringenin can effectively inhibit the progression of MASH, reversing adverse changes by mitigating cellular damage, lipid deposition, and reducing oxidative stress in the liver. Furthermore, the authors speculate that naringenin may inhibit the development of steatosis and inflammation by lowering levels of lysophosphatidylethanolamine (LPE) [83]. The consumption of bioactive compounds, including narginine, can favorably influence intestinal bacterial growth and gene expression and increase the abundance of bacteria responsible for SCFA production [84,85]. The supply of orange juice to a group of 10 women for 2 months positively modulated the composition and metabolic activity of the microbiota, increasing the population of fecal *Bifidobacterium* spp. and *Lactobacillus* spp. In addition, changes in blood biochemical parameters such as a reduction in LDL and glucose levels and an improvement in insulin sensitivity were observed [85]. Results from a randomized, double-blind, placebo-controlled clinical trial by Namkhah et al. showed that the daily administration of 200 mg of naringenin for 4 weeks had a beneficial effect on lipid profile and the percentage of MAFLD grade as an indicator of the severity of liver steatosis [86]. Furthermore, according to Naeini et al., naringenin supplementation may be a promising strategy for the treatment of cardiovascular complications in patients with MAFLD [87].

The current scientific reports on the simultaneous effects of naringin and naringenin on microbiota composition and liver health are presented in Table 2.

### 2.4. Resveratrol

Resveratrol is a polyphenol known for its powerful antioxidant, anti-inflammatory, anti-cancer, neuroprotective, and anti-diabetic properties. In addition, it is believed to be an aid in the treatment of cardiovascular disease or obesity. The main sources of this compound are blueberries, grapes, peanuts, and red wine [88,89]. A recent meta-analysis of preclinical and clinical studies demonstrated that resveratrol at a dose of 50–200 mg/kg and a time interval of 4–8 weeks showed an effect in alleviating MAFLD in preclinical studies [90]. It is likely that the hepatoprotective effect of resveratrol is related to inhibition of the NF-κB pathway and activation of the SIRT-1 (sitrulin 1) and AMPK (activated protein kinase) pathways, although the results of clinical trials are still inconclusive [90,91].

Asghari et al. found that resveratrol supplementation could affect weight loss in patients with MAFLD, but the benefits of calorie restriction were greater and were associated with reductions in body weight, BMI, waist circumference, and ALT and AST activity [92]. Different results were obtained by Chen et al. Supplementation with resveratrol at a dose of 150 mg twice daily for 3 months resulted in a reduction in AST, ALT, glucose, LDL cholesterol, TC, and the insulin resistance index [93]. Nevertheless, there are also data from clinical trials that do not support the efficacy of RSV supplementation in the treatment of MAFLD [94,95,96,97,98,99,100].

#### Effect of Resveratrol on the Liver, Metabolic Status, Inflammation, and Gut Microbiota

Resveratrol (RSV) and its derivatives appear to also modulate the composition of the intestinal microbiota and to affect the intestinal barrier function and intestinal tight junction [101,102]. Cai et al. showed that resveratrol improves intestinal barrier function and attenuates intestinal permeability and inflammation in mice with diabetic nephropathy. Furthermore, the supply of resveratrol reversed dysbiosis characterized by a low abundance of the genera *Alistipes*, *Alloprevotella*, *Bacteroides*, *Odoribacter*, *Parabacteroides*, and *Rikenella* [103]. Interestingly, resveratrol has been shown to alleviate liver fibrosis precisely by affecting the composition of the microbiota. Li et al. observed that this could occur by inhibiting the growth of *Staphylococcus_xylosus* and *Staphylococcus_lentus* [104]. Nevertheless, the evidence for this is still inconclusive. In a study in an animal model, resveratrol administration alleviated steatosis and hepatitis induced by high-fat and high-fructose diets. However, no differences in microbiota composition were observed, although the abundance of *UBA-1819* was associated with a lower liver weight in animals that received resveratrol concurrently [105], although an earlier observation by this team showed a greater presence of *Lactococcus* and *Blautia* bacteria in the microbiome of animals receiving resveratrol [106]. Similarly, Du et al. also observed differences in the gut microbiota of mice with MAFLD even with a low RSV supply. At the type level, the predominant bacteria were *Firmicutes*, *Bacteroidetes*, and *Actinobacteria*. A tendency was also observed to promote the growth of *Erysipelotrichaceae* and inhibit the growth of *Ruminococcaceae*. It has also been shown that RSV can restore the expression of genes related to lipid metabolism to normal levels and repair the damaged insulin signaling pathway [107]. In a study by Wang et al., resveratrol supply attenuated weight gain in HFD-fed mice and inhibited fat accumulation without affecting energy intake. Resveratrol treatment improved glucose tolerance and insulin resistance. Improvements in intestinal permeability, intestinal villi length and density, and effects on uniform distribution were also observed. Increased mRNA expression of tight junction proteins has also been reported [108].

Current scientific reports on the simultaneous effects of resveratrol on microbiota composition and liver health are presented in Table 3.

### 2.5. Quercetin

Quercetin is an agglucon or aglycone that does not contain any carbohydrate molecules in its structure. Rich dietary sources of quercetin include capers, rocket, dill, coriander, fennel, juniper berries, elderberries, field poppy, bee pollen, and muskmelon [109]. Quercetin is characterized by its anti-inflammatory, anti-cancer, and antiviral properties and has been used in the treatment of age-related diseases, diabetes, cardiovascular disease, and hypertension [110]. Recent data show that it can prevent the onset of Alzheimer’s disease by inhibiting amyloid β aggregation [111]. The molecular structure of quercetin allows it to eliminate oxygen-free radicals and produce metal-chelating compounds [112]. In addition, quercetin may have a role in the reduction in abdominal obesity through its effects on the gut microbiota [113].

#### Effect of Quercetin on Liver, Metabolic Status, Inflammation, and Gut Microbiota

There are a number of data supporting the effect of quercetin on the development of liver disease through various mechanisms. Analysis by Zhao et al. showed that quercetin can inhibit liver inflammation primarily through NF-κB/TLR/NLRP3 and can also reduce phosphatidylinositol 3-kinase (PI3K)/nuclear factor erythroid 2-related factor 2 (Nf2)-dependent oxidative stress. In addition, it can activate mTOR in autophagy and inhibit the expression of apoptotic factors associated with the development of liver diseases. Interestingly, the results indicate that quercetin has an effect on the regulation of PRAR, UCP, and protein perlipin (PLIN) factors through activation of brown adipose tissue in liver steatosis [114]. Increased UCP1 expression is associated with increased WAT browning and BAT activity, by activating the AMPK/PPARγ pathway, which can help prevent obesity and metabolic complications [115]. Pei et al. observed that quercetin could upregulate UCP1 gene expression in HFD-fed mice, leading to the bronzing of retroperitoneal WAT and weight reduction. Quercetin also decreased the ratio of *Firmicutes* to *Bacteroidetes* and increased SCFA production [116].

In a study by Feng et al. involving laying hens in which mucosal damage, necrosis, and exfoliation were induced, quercetin markedly increased total antioxidant capacity and glutathione peroxidase activity. Quercetin supplementation restored goblet cell density, mucin2 expression levels, and Claudin1 and Occludin mRNA expression in the intestinal mucosa. The expression of IL-1 β i TLR-4 in the intestinal mucosa and an increase in bacteria responsible for SCFA production, such as *Negativicutes*, *Phascolarctobacterium*, *Megamonas*, *Prevotellaceae*, *Bacteroides*_*salanitronis*, and *Selenomonadales*, were also observed [117]. Transplantation of the gut microbiota of donors who respond to the HFD diet predisposed germ-free mice to MAFLD and the supply of quercetin had a protective effect on the development of metabolic changes by blocking the gut–liver axis. Moreover, a protective role for *Akkermansia* genus bacteria was observed in the development of MAFLD [118]. In a study by Juárez-Fernández et al., the simultaneous administration of *Akkermansia muciniphila* and quercetin resulted in steatosis remission, associated with modulation of liver lipogenesis. Synbiotic supply increased the plasma levels of unconjugated hydrophilic bile acids and increased liver expression of BA synthesis and transport genes [119]. Zhao et al. also confirmed the existence of the gut–hepatic axis. The supply of *Lactobacillus plantarum NA136* corrects gut microbiota disorders caused by a fat- and fructose-rich diet by strengthening the intestinal barrier and reducing inflammation in the liver [120].

A meta-analysis of 16 case–control studies in recent years showed that quercetin supplementation is associated with lower levels of total cholesterol (TC), low-density lipoprotein (LDL), and C-reactive protein (CRP) in patients with metabolic syndrome and related disorders [121]. The results of in vivo and in vitro studies indicate that quercetin and its derivatives exert hepatoprotective effects against MAFLD by regulating oxidative stress and inflammation, and also through changes in the gut microbiota [122]. In Porras et al.’s study, quercetin supplementation reduced intrahepatic lipid accumulation through the modulation of lipid metabolism gene expression, cytochrome P450 2E1 (CYP2E1)-dependent lipoperoxidation, and associated lipotoxicity. Thus, it reduced insulin resistance and MAFLD activity and reversed dysbiosis [123]. The results of a randomized, double-blind, placebo-controlled, crossover study showed that a 500 mg dose of quercetin for 12 weeks was associated with reductions in intrahepatic lipids, body weight, and BMI [124].

The result of a study by Abd El-Emam et al. showed that the administration of quercetin liposome (QR-lipo) can protect against Co-Amox-induced hepatotoxicity. The combined administration of Co-Amox and QR-lipo significantly improved antioxidant status. Furthermore, a protective effect of QR-lipo against antibiotic-induced intestinal dysbiosis was observed [125]. Combination therapy with quercetin and anti-PD-1 antibodies altered necrosis, fibrosis, and PD-L1 expression in liver tissues, thus reducing the tumor microenvironment of HCC in mice, while upregulating the gut microbiota and macrophage immunity. An increase in the abundance of *Firmicutes*, *Actinobacteria*, and *Verrucomicbiota* has also been reported at the type level, as well as an increase in *Dubosiella* and *Akkermansia* at the genus level [126]. Similarly, in a study by Xie et al., the administration of buckwheat quercitin to rats at a dose of 200 mg/kg bw had a positive effect on the gut microbiota and significantly reduced body weight, the liver index, and lipid levels, thus reducing oxidative stress and positively influencing the repair of damaged liver [127].

The current scientific reports on the simultaneous effects of quercetin on microbiota composition and liver health are presented in Table 4.

## 3. Long-Term Use Safety of the Flavonoids Discussed in the Review

According to Soleimani et al., silymarin is safe for human consumption at therapeutic doses and can be well tolerated, even at a high dose of 700 mg given three times daily for 24 weeks [128]. Toxicity studies in animals treated long-term with silymarin confirmed its high tolerability and studies with humans provided data on the most common side effects such as headaches and itching, which can occur at high doses [129].

The threshold for no observed adverse effects from genistein is considered to be 50 mg/kg/day, based on the presence of mild liver effects at the high dose of 500 mg/kg/day [130]. Genistein is generally considered to be well tolerated and safe. Minimal toxicity may include symptoms of nausea, leg cramps, or tenderness and may occur at a dose of 16 mg/kg body weight [131].

According to Rebello et al., naringenin doses of 150–900 mg are safe in healthy adults, and serum concentrations are proportional to the dose administered. Naringenin metabolites present in the circulation are eliminated within 24 h [132]. Toxicity studies have confirmed the safety of naringenin in animal models, suggesting its potential for safe administration to humans. However, controlled clinical trials are needed to confirm its safety for long-term use [133].

Current data suggest that quercitin supplementation is safe, and adverse effects in humans have been rare in the studies that have been conducted. However, some results from animal studies suggest that its long-term use may have nephrotoxic effects, especially in cases of pre-existing kidney damage [121].

Randomized clinical trials suggest that resveratrol supplementation is safe in both adults and adolescents [94,134]. Short-term resveratrol supplementation at doses of 300 mg/day and 1000 mg/day does not adversely affect blood chemistry and is well tolerated by overweight older adults [135]. RSV has shown beneficial effects on cognitive function in the elderly, but at high doses increased some biomarkers of cardiovascular risk (at a dose of 1000 mg for 90 days). Therefore, it is considered that optimal dosing, long-term effects, and potential drug interactions need to be investigated in well-designed randomized clinical control trials [135,136].

## 4. Conclusions

Liver diseases, including MAFLD, are a serious health problem, leading to high mortality rates and the risk of developing cirrhosis and hepatocellular carcinoma. Flavonoids, natural compounds present in a variety of plant products, have shown promising therapeutic properties in the context of liver health. Numerous studies show that flavonoids can improve the lipid profile of the liver, reduce inflammation and oxidative stress, and modulate the composition of the gut microbiota. In particular, constituents such as silymarin, genistein, quercetin, and narginine/nirginine have shown the ability to reduce lipid accumulation in the liver, improve glucose metabolism, and alleviate inflammation.

Additionally, their effects are often associated with improved intestinal barrier function and the modulation of gut microbiota activity, which may contribute to protection against MAFLD progression. Supplementation and dietary changes based on flavonoid sources can lead to significant improvements in health parameters for patients with liver disease. As a result, understanding the role of flavonoids in the management of MAFLD opens new opportunities for nutritional therapies that can be customized to optimize liver health and reduce the risk of severe complications associated with chronic liver disease. Still, most of the scientific evidence comes from preclinical studies, which is why well-designed long-term clinical trials are so important.

## Figures and Tables

**Table 2:** Simultaneous effects of narginine and nirginine on the liver, metabolic state, inflammation, and gut microbiota.

Name of the Active Compound	Results of the Study	Reference
Animal studies		
Naringin	reduction in serum lipids, ALT, AST, glucose, hsCRP, and LPS; attenuation of liver inflammation; negative correlation of TC, HDL-C, and LDL-C parameters with *Allobaculum*, *Alloprevotella*, *Butyricicoccus*, *Lachnospiraceae*_*NK4A136_group*, *Parasutterella*, and *uncultured*_*bacterium*_*f_Muribaculaceae*; *Campylobacter*, *Coriobacteriaceae_UCG-002*, *Faecalibaculum*, and *Fusobacterium* were positively correlated with serum lipids; reduction in ALT levels; no differences in AST levels; restoration of normal serum glucose and hsCRP levels.	Mu et al. (2020) [81]
Naringenin	less weight gain; reduction in fat mass and liver mass; reduction in serum TC and TG levels; reduction in inflammatory factors (TNF-α, IL-6 and inflammatory marker F4/80) and MPO activity; inhibition of infiltration of colon macrophages and dendritic cells; improvement in intestinal permeability; alleviation of the decrease in colonic protein levels (ZO-1, claudin-1, and Occludin); increase in claudin-2 levels; inhibition of the downward trend induced by HFD in gene expression; decreasing the abundance of *Proteobacteria* and increasing the abundance of *Bacteroidetes*; increasing the levels of *Epsilonproteobacteria* and *Deltaproteobacteria*; inhibition of cecal bacterial overgrowth.	Yu et al. (2023) [82]
	lowering TC and TG levels; improving AST, ALT, MDA, and GSH levels; increasing KN-93 levels and inhibiting hepatic stellate cell proliferation; restoring favorable levels of *Akkermansia* and *Enterobacter*; increasing the [*Eubacterium*] *nodatum* group.	Cao et al. (2023) [83]

Abbreviations: AST—aspartate aminotransferase, ALT—alanine aminotransferase, hsCRP—high-sensitivity C-reactive protein; LPS—lipopolysaccharide; MDA—malondialdehyde; GSH—glutathione; TC—total cholesterol; TG—triglycerides; HDL-C—high-density lipoprotein; LDL-C—low-density lipoprotein cholesterol; TNF-α—tumor necrosis factor-α, IL-6—interkulin-6; MPO—myeloperoxidase; HFD—high-fat diet.

**Table 3:** Simultaneous effects of resveratrol on the liver, metabolic state, inflammation, and gut microbiota.

Name of the Active Compound	Results of the Study	Reference
Animal studies		
Resveratrol	lowering liver weight; lowering serum TC and non-HDL cholesterol levels, and increasing serum HDL cholesterol levels; reduction in AST and ALT levels; no significant changes in the gut microbiota.	Milton-Laskibar et al. (2022) [105]
	downward trend in ALT, AST, TG, cholesterol, and LDL-C levels and body weight; reduction in TG levels and fasting blood glucose and HOMA-IR insulin levels; increased relative abundance of *Actinobacteria*; increased levels of zo-1 and occludin protein and zo-1 gene expression; decreased levels of signaling molecules; TLR4 and MyD88 and inflammatory IL-1 and TNF-α.	Du et al. (2021) [107].
	complete inhibition of HFD-induced MDA, ROS, and GSH-Px, and restoration of SOD and T-AOC activity in the liver of HFD-fed mice; restoration of normal HDL, LDL, ALT, and AST levels; reduction in TC and TG levels (including lower liver concentrations); improvement in fasting glucose and insulin values; increased mRNA expression of tight junction proteins (ZO-1, ZO-2, and occludin) and interstitial adhesion molecule A (JAM-A); restoration of the expression of TFF3, Muc1, Relmβ and Reg3γ; increase in relative abundance of *Allobaculum*, *Bacteroides*, and *Blautia* in mice fed an HFD diet; increased mRNA expression of the cytokine IL-10 and decreased mRNA expression of pro-inflammatory cytokines such as IL-16, IL-6, and IL-1β; increased mRNA expression of genes related to mitochondrial function, such as NRF-1, Tfam, UCP3, PGC-1α, and SIRT1.	Wang et al. (2020) [108]
	decreased collagen deposition in the liver; decreased expression of α-SMA and Collagen I in liver tissues; inhibition of ZO-1 loss; partial improvement in ALT, AST, and ALP.	Li et al. [45]

Abbreviations: TC—total cholesterol; HDL—high-density lipoprotein; AST—aspartate aminotransferase, ALT—alanine aminotransferase; TG—triglycerides; LDL-C—low-density lipoprotein cholesterol; HOMA-IR—homeostasis model assessment of insulin resistance; TFF3—trefoil factor family protein 3; Relmβ—resistin-like molecule β; NRF-1—nuclear respiratory factor 1; Tfam—mitochondrial transcription factor A; UCP3—uncoupling protein 3; PGC-1α—peroxisome proliferator-activated receptor gamma coactivator 1-alpha; SIRT1—sirtuin 1; α-SMA—α-alpha-smooth muscle actin; TLR4—toll-like receptor 4; MDA—malondialdehyde; SOD—superoxide dismutase; T-AOC—total antioxidant capacity; HFD—high fat diet; ROS—reactive oxygen species; ZO-1—zonula occludens-1; ZO-2—zonula occludens-2; ALP—alkaline phosphatase.

**Table 4:** Simultaneous effects of quercetin on the liver, metabolic state, inflammation, and gut microbiota.

Name of the Active Compound	Results of the Study	Reference
Animal studies		
Quercitin	reduction in ALT and AST concentrations; no effect on albumin concentrations was noted; decrease in MDA levels and increase in GSH, CAT, and TAC levels; activation of IL-10 gene expression; increased expression of SIRT1 and Nrf2; decreased expression of pro-inflammatory liver factors, including IL-6, IL-1β, TNF-α, NF-κB and iNOS, reduction in Keap1 levels; increase in the abundance of *Bifidobacterium*, *Bacteroides*, and *Lactobacillus*; reduction in *Clostridium* and *Enterobacteriaceae*.	Abd El-Emam et al. (2023) [125].
	stunted weight gain; lowering liver index; lowering TC, TG, and LDL-C levels and increasing HDL levels; alleviation of lipid peroxidation (higher levels of GSH-PX, lower CHE levels); lowering the levels of NF-kB, TLR4; lowering the ratio of *Firmicutes* to *Bacteroidetes*; reduction in the abundance of proteobacteria; reduction in the growth of *Lachnospiraceae*; increased the amount of relative abundance of *Oscillospiraceae*, *Ruminococcaceae*, *Christensenellacea*, and *Eubacterium*_co-prostanoligenes_group.	Xie et al. [127].

Abbreviations: AST—aspartate aminotransferase, ALT—alanine aminotransferase; MDA—malondialdehyde; GSH—glutathione; CAT—catalase; TAC—total antioxidant capacity; IL-10—interleukin 10; SIRT1—sirtuin 1; Nrf2—nuclear factor erythroid 2-related factor 2; IL-6—interleukin-6; IL-1β—interleukin 1β; TNF-α—tumor necrosis factor α; NF-κB—nuclear factor kappa B; iNOS—inducible nitric oxide synthase; TC—total cholesterol; TG—triglycerides; LDL-C—low-density lipoprotein cholesterol; GSH-PX—plasma glutathione peroxidase; CHE—cholinesterase; TLR4—toll-like receptor 4.
